# Instability of Personality Traits of Teachers in Risk Conditions due to Work-Related Stress

**DOI:** 10.3390/bs10050091

**Published:** 2020-05-13

**Authors:** Ruggero Andrisano Ruggieri, Anna Iervolino, PierGiorgio Mossi, Emanuela Santoro, Giovanni Boccia

**Affiliations:** 1 Department of Human, Philosophical and Educational Science, University of Salerno, 84084 Fisciano (Sa), Italy; annaiervolino@hotmail.it; 2 National Labor Inspectorate, Agency of Minister of Labour, 72100 Brindisi, Italy; pmossi@gmail.com; 3 Department of Medicine and Surgery, University of Salerno, 84084 Fisciano, Salerno, Italy; esantoro@unisa.it (E.S.); gboccia@unisa.it (G.B.)

**Keywords:** stress, Big Five, personality, work-related stress, pathology

## Abstract

The following study aims to verify whether psychosocial risk conditions determine a variation in personality traits. The sample consisted of 301 teachers, comprising 84 men (27.1%) and 217 women (72.9%). The Big Five Questionnaire (BFQ) was used to measure personality traits, while the Organizational and Psychosocial Risk Assessment (OPRA) questionnaire was used to measure psychosocial risk. The ANOVA results notice the change of BFQ traits. These are significant (Extraversion = 0.000; Agreeableness = 0.001; Neuroticism = 0.000; Openness = 0.017), with the exception of the Conscientiousness trait (Conscientiousness = 0.213). The research supports the approach of seeing personality as the result of the interaction between the individual and the environment; this position is also recognized by work-related stress literature. Stress conditions can lead to a change in the state of health and possibly determine the onset of work-related stress diseases. In the future, it would be useful to start a series of longitudinal studies to understand in greater detail the variability of personality traits due to changes in the Risk Index.

## 1. Introduction

Teaching is a challenging and undoubtedly difficult profession [1] the current literature shows that it is a high-stress occupation [2,3,4,5]. It is often described as being made up of negative emotions characterized by anger, anxiety, frustration or depression, fatigue and difficulty concentrating [4,6,7,8].

In light of these descriptions, numerous studies have analysed different relationships, such as those between stress and the coping strategies useful to address it [9,10]; stress and cardiovascular, musculoskeletal and metabolic system diseases [11,12,13]; and burnout syndrome [14,15].

Many studies have investigated the individual differences as useful characteristics through which to understand the outcomes of stress and the pathologies related to it. The constructs of “locus of control” and “A and B personalities”, the Big Five traits and self-efficacy theory [16,17,18,19,20,21,22,23,24,25,26,27,28] represent attempts to understand those individual properties as either vulnerabilities to disease or, on the other hand, methods of resilience.

However, this working approach is significantly conditioned by a classic tradition which, in some ways, is affected by the absence of preventive strategies in the workplace, since it focuses on the detection of nosographic categories that can be framed in diagnostic manuals such as the DSV and ICD-11. In other words, this approach proposes, through the concept of diagnosis, an individualistic and contextual perspective in the development of a pathology or, more generally, of a condition of malaise [29]. It is impossible to apply primary prevention, and it is therefore necessary to operate solely according to a secondary or tertiary prevention logic.

In contrast, current research on work-related stress has proposed a paradigmatic reversal due to its interest in organizational well-being and health in the workplace [30,31,32,33]. These studies have focused on the work risk factors that determine a fertile ground for pathology development, thus allowing for the adoption of a primary prevention logic.

In Italy, this preventive operation was made possible through Legislative Decree no. 81/2008 and the circular issued by the Ministry of Labour and Social Policies of November 18, 2010; these items made the assessment of occupational stress compulsory in Italy [34,35,36,37], as set out by the Framework Agreement on Work-Related Stress (October 8, 2004). According to the Legislative Decree 81/2008, work-related stress is defined as an imbalance that can occur when the worker does not feel able to respond to job requests.

Not all the signs of stress at work are work-related, since stress is caused by various factors specific to the context (i.e., decision-making autonomy and control; role in the organization; interpersonal conflicts at work; ambivalence regarding performance and requests; career evolution and development, etc.) as well as the content of the job (i.e., matching between workers’ skills and professional requirements; workloads and rhythms; work environment and equipment; shifts and working hours). These regulatory aspects have direct correspondence with the findings within the literature on the subject. The question-control model was developed by Karasek (1979) [38] to clarify why individual job conditions do not lead to the manifestation of stress-related diseases but their interaction can [23,39]. It was founded on two principal factors: (a) job demand (the complexity and load of the work), which is considered as a physical and mental effort required to perform a specific assignment, and (b) job control (decision-making autonomy), linked to the ability to carry out the consigned task [17]. It has been highlighted how the concurrent presence of elevated levels of job demands and poor levels of job control is often associated with a general feeling of discomfort, illness or unease, along with a low job satisfaction [23]. Other studies have considered psychosocial factors that are strongly associated to the work environment, such as the instrumental and social-emotional support received from colleagues and superiors [21,40]. The sources of stress in terms of pressures from the environment that affect individual-level psychophysical states were defined by Cooper and Marshall (1976) [41]. This results in a reduced work performance, absenteeism and poor job satisfaction [23,42]. There are five categories: intrinsic sources at work (e.g., lighting, noise), role-related factors, career development (CD), work relationships (relationship difficulties) and, finally, the structure and organizational climate (constraints on decision-making and budget, etc.).

The most interesting aspect of Cooper’s (1986) [43] model is the introduction of a work–life balance (WLB). The close relationship between work and private life was recognized by Gatrell and Cooper (2008) [44]. They highlighted how the stress response can depend on factors related to the family context, but also on the ease/difficulty of combining personal-family life and professional requests [45,46,47,48,49]. Job satisfaction is a condition of well-being at work that promotes a positive attitude towards employees, thus increasing their availability in the working environment and improving productivity [50,51]. Job satisfaction is also associated to the characteristics of the work environment rather than individual variables [51,52,53,54].

The parameters used to evaluate the working environment include interpersonal conflicts, complaints, overtime, letters of recall, absenteeism and accidents at work. They can all be considered indicators of the possible development of work-related stress [34,35,36,37], as well as a manifestation of organizational malaise [55,56].

For this reason, the abovementioned law establishes that risk assessment should be identified through the preparation of a methodological path to allow for the correct identification of risk factors due to stress at work. This approach drives the planning and implementation of measures aimed at either eliminating or, when elimination is not possible, minimizing the risk factors, i.e., *work content* and *work context* [57,58,59,60,61,62]. For some authors, however, even if it may be true that we are able to define physical risks with a good approximation, the same is not true of psychosocial risk (i.e., work-related stress), since it is not possible to regulate only the causal relationship between danger and damage. Psychosocial risk is discussed as the result of the interaction between the work context and the subjective characteristics of the worker [63]. Others, however, have analysed the relationship between stress and personality traits [56,64,65].

Focusing on the latter approach, Cattel described personality as a cognitive-behavioural model that is stable over time and in different situations [15,66]; the Big Five is the most widespread and widely used model in the study of personality, deriving from the studies carried out by Cattel. This model reduces personality to five traits: extroversion, open-mindedness, amiability, conscientiousness and neuroticism [15,67,68]. Some studies have tried to understand if and which personality traits can be considered predictors of stress [64,69,70,71,72,73,74].

Therefore, the aim of this study is to analyse in greater detail the relationship between personality and work-related stress. It also verifies whether personality traits change under the conditions of psychosocial risk or if the conditions of psychosocial risk are able to determine a variation in personality traits. The hypotheses are as follows:

(a) H_o_ personality traits are stable and invariant with respect to psychosocial risk
(b) H_o_ personality traits vary with psychosocial risk conditions.

## 2. Materials and Methods

The questionnaires were administered anonymously according to a collective and individual data collection method in various higher education institutions in the city of Salerno, resulting in a non-probabilistic sample of 301 teachers consisting of 84 males (27.1%) and 217 females (72.9%). Most of them are hired on permanent contracts, part-time (56.5%) or full-time (38.2%), and their length of service is mostly 25 years (55.5%); the remaining teachers (45%) have less than 25 years of service. They are divided into scientific (79.4%), commercial/economic (7.3%) and classical (13.3%) high schools. 53.5% teach technical-scientific subjects (mathematics, physics, chemistry, etc.) and the others (46.5%) teach humanities (literature, history, second language, etc.).

The survey administration took place after the signing of the data release forms. To evaluate the link between the variables, we used the linear correlation statistical technique (Pearson coefficient), which allowed to establish not only the relationships between the variables but also their intensity [75]. In the next phase, the ANOVA statistical technique was used to exclude the possibility that the relationships between the variables were due to chance, while also establishing the relationships between personality traits and the psychosocial risk of work-related stress conditions. The risk of work-related stress was the independent variable, and the personality traits were the dependent variables. Statistical Package for Social Science v20 software (SPSS) was run [76].

The risk of occupational stress and personality traits were evaluated through an Organizational and Psychosocial Risk Assessment (OPRA) questionnaire [23] and a Big Five Questionnaire (BFQ), respectively.

### 2.1. OPRA

The Italian Academy developed a multifactorial questionnaire, known as OPRA, to evaluate the presence of psychosocial risk factors and work-related stress conditions in accordance to both the requirements of Legislative Decree no. 81/08 and the discoveries of the current literature. The questionnaire has three parts, each one evaluating diverse characteristics of the job experience. Each section uses a Likert-type 5-point scale (ranging from never to always) and has the following three indices.

### 2.2. Risk Index (RI)

It has five items that evaluate the general feeling of discomfort, illness or unease resulting from a condition of low identification with the group and organization due to low job satisfaction, poor confidence in the organization and the desire to leave the workplace (e.g., How confident are you in the ability of the organization to best use the results of this survey to improve the current work conditions? What is your level of satisfaction in your current job?). It has a Cronbach’s alpha value of 0.71.

### 2.3. Inventory of Source of Risk (ISR)

ISR has 65 items distributed over different factors to evaluate the sources of occupational stress that may cause distress or discomfort. There are the following subscales: (1) Culture and Organization; (2) Role; (3) Career Development; (4) Autonomy at work; (5) Work Life Balance; (6) Environment and Safety; (7) Workload; (8) Working Time; (9) Quality of Relationship. It results in a classification of subjects into risk categories (negligible, medium low, medium high, high and critical) and has a Cronbach’s alpha value of 0.71.

### 2.4. Mental and Physical Health (MPH)

It is a two-dimensional scale, with 16 items evaluating the frequency of physical and psychological disorders. It gives an aggregated score that estimates the effects of stressful working situations (e.g., neck pain, difficulty falling asleep or disrupted sleep, excessive tiredness, headaches) and has a Cronbach’s alpha value of 0.90.

### 2.5. Big Five Questionnaire (BFQ)

The BFQ [77] is an Italian standardized psychometric tool derived directly from the NEO Personality Inventory (NEO-PI) of Costa and McCrae. The latter was also used for correlation analysis to establish the validity of the BFQ. It consists of 132 items scored on 5-point Likert scales that provide the degree of agreement with each item. The 5 personality traits are as follows. (1) Energy (E): this dimension evaluates the quality and intensity of interpersonal relationships, the level of activity, the need for stimulation and the ability to feel joy. High scores on this scale refer to active, sociable and talkative behaviours. This is called Extroversion in the Costa and McCrae model (α 0.81). (2) Agreeableness (A): this dimension evaluates the quality of interpersonal orientations in an uninterrupted series of thoughts, feelings and actions, ranging from compassion to antagonism. It corresponds to Pleasantness in the Costa and McCrae model (α 0.73). (3) Conscience (C): this dimension evaluates the degree of organization and perseverance of an individual. It corresponds to Conscience in the Costa and McCrae model (α 0.81). (4) Emotional stability (S): this dimension evaluates the presence of a restless, nervous and a more or less unstable emotional state, as well as the ability to control emotions and behaviours in conflict situations. It corresponds to the trait of Neuroticism in the Costa and McCrae model (α 0.90). (5) Mental openness (M): this dimension evaluates the proactivity and openness towards spontaneous and novel experiences and the pleasure of exploring what is unfamiliar; essentially, it is the attitude towards novelty. It corresponds to Openness to Experience in the Costa and McCrae model (α 0.75).

## 3. Results

We initially analysed the correlation between the risk index (RI), the inventory of risk sources (ISR) and the inventory of psychophysical health (MPH) with the five personality traits. The minimum significance level adopted was 0.05 (two-tailed test). As shown in Table 1, the features of the BFQ are in most cases correlated with the scales of the ISR and those of the MPH, mostly in a negative way. As the scores of the variables relative to the ISR and MPH increase, there is a decrease in the scores of the traits of the BFQ.

In contrast, we have a positive correlation between the following ISR and BFQ variables: (a) between B1 and the traits of the BFQ, with the exception of the Openness trait, which does not appear to be significant (0.102); (b) between B4 and all sections of the BFQ; and (c) between B6 and the sections of the BFQ, with the exception of the Openness trait, which does not appear to be significant (0.099). The correlation between B8 and the BFQ does not appear to be significant except for the Neuroticism trait (−0.1 5d40). Furthermore, there is an absence of correlation between the Conscientiousness trait (−0.140) and B7 (−0.062), B8 (0.046) and C2 (−0.078).

**Figure 1:**
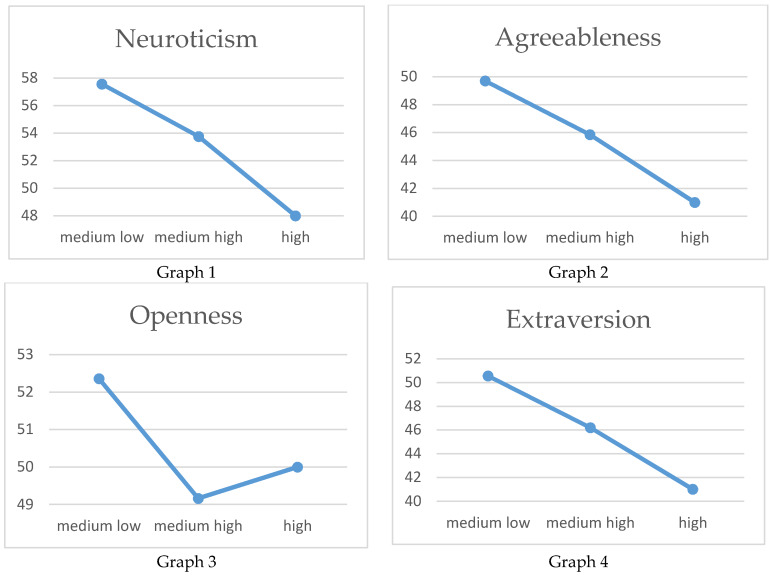
Graphical representation of the data averages in Table 2.

The variance analysis through the ANOVA test shows how all the data are significant (Extraversion = 0.000; Agreeableness = 0.001; Neuroticism = 0.000; Openness = 0.017), with the exception of the Conscientiousness trait (Conscientiousness = 0.213) (Table 2). There is a graphical representation of the results in Table 2 and in Figure 1 (Graphs 1, 2, 3, 4).

The multiple comparison of the averages performed, i.e., Fisher’s least significant difference (LSD), shows that significance is evident in the relationship between the RI (medium high and high) and the traits of Extraversion, Agreeableness and Neuroticism. In contrast, the significance between Openness and the RI is evident between medium low and medium high as well as between medium high and high as it showed in Table 3.

## 4. Discussion

Personality is a complex organization of ways of being, knowing and acting that ensures unity, coherence and continuity, stability and planning in the individual’s relationships with the world [78]. The definition of personality has always been a field of study in psychology, that can be clearly defined as personality psychology. Personality is therefore theorized and studied by means of real taxonomies. By using factorial analysis for the study of personality, an attempt was made to reduce its phenomenal manifestations to their fundamental latent dimensions [66].

Concerning the term traits, it is customary to indicate those characteristics of the personality that are mostly considered to be of genetic origin [79] and therefore difficult to modify, since they influence human behaviour in a stable way.

From this perspective, the theory of the BFQ is used as a method for studying personality in its structural aspects, i.e., in terms of the architecture of its dispositions. These guidelines share the assumption that there is a latent structure of traits or basic provisions that accounts for behavioural and psychological manifestations that appear to be stable and long-lasting, and therefore independent of situations and contexts, since they are based on genetic aspects [80].

However, this research seems to support the studies that have criticized the BFQ model because it considers personality as a phenomenon in itself and not as a result of individual dynamics with the environment [81].

Personality is caged within a rigid scheme, in which behaviour becomes something predictable because it originates from the personality, which is considered stable in all situations.

In contrast, clinical psychology has embraced the concept of a different personality, i.e., a dynamic nature that is therefore unstable, which is created through strong interactions between the individual and the context. This instability finds expression in the concept of state within the test measurement systems, but above all in the concept of diagnosis, which is always enriched with the criteria of onset as the disease progresses [82]; in other words, mental illness is considered to be a state, since healing is possible. For example, Spielberger et al. developed the State-Trait Anxiety Inventory (STAI) (1970) [83]. This is the best-known and most widespread tool in this field; it is divided into two sub-scales, namely the STAI T-Anxiety Scale and the STAI S-Anxiety Scale, which explore anxiety traits and anxiety states, respectively. In 1983, based on the experience gained over 10 years of use, a revision of the STAI, Form Y, was published, which is capable of distinguishing the two types of anxiety more clearly. It is evident that, in the distinction between anxiety traits and anxiety states, aspects of personality and behaviour dynamics are captured due to the conditions in which the subject is operating [84].

This dynamism has also been recognized by studies on work-related stress, due to the weight that the context and work content factors can have for the purpose of generating pathologies that are compatible with stress conditions. In our opinion, our research work results should be interpreted from this perspective, especially the significance of the RI detected by the ANOVA.

The change in the BFQ personality trait scores denotes, for example, a drop in the Extroversion scores due to an increase in the RI. Therefore, it appears counterintuitive that in conditions in which job dissatisfaction and the desire to change jobs increase, detachment and withdrawal from work activities occur. The same happens for the Agreeableness trait behaviours, demonstrating a dizzying reduction of polite and tolerant behaviours due to taking on cynical, suspicious and uncooperative behaviours. Therefore, in these working conditions, it is clear that behaviours drive the determination of emotional stability loss (Neuroticism) towards anxious and stressful behaviours. The same happens for the trait of mental Openness, where the original and creative behaviours are replaced by conformism and a refusal to experiment. These variations in scores appear strongly compatible with the stress condition.

Future research is required to assess emotionally unstable personality traits among teachers, such as the borderline personality trait that is characterized by mood swing [85], impulsiveness, interpersonal problems and cognitive impairment [86]. Such personality traits can lead to work-related stress.

## 5. Conclusions

Our research confirms the literature data showing how stress conditions can determine a change in health conditions and possibly the onset of related work-related stress diseases.

However, this contextual and dynamic dimension also seems to affect the characteristics of the personality of the subjects that have previously been considered stable.

This type of consideration is not unrelated to clinical work: note, for example, that in the event of mobbing and/or bossing in the workplace, legal psychology has identified systems for assessing mental damage [87], with the aim of identifying the variation of the subjects’ personalities and the causal link with the violence suffered in the workplace.

This operation is not necessarily attributable to a diagnostic category; therefore, it relates not only to the onset of disease but also and above all to the referable change in behaviours that highly impacts the lifestyle of the subjects examined [88,89].

Our study is not necessarily attributable to the onset of disease. It would be useful to start a series of longitudinal studies to better understand the variability of personality traits due to changes in the RI.

This study has several limitations. The sample is certainly not representative, as it is not sufficiently extensive from a statistical point of view. The results are introductory and should be confirmed in other contexts. To increase the generalizability of data, longitudinal studies could be used taking into account these types of study and exposed to historical and maturation effects, e.g., missing values due, for example, to the turn over, as well as a sensitization to the detection tools that invalidate the reliability of the data. Some aspects of the possible weight of the sex variable should be further investigated. Usually, sex appears to be a mediator of dimensions such as resilience, coping, etc. A deeper analysis of the characteristics of respondents could have improved the quality of the obtained results. Greater attention to the construction of the characteristics of the sample could have improved the quality of the findings. For instance, this study classes personality based on the BFQ. There are other, more clinically-oriented classifications, such as borderline personality traits. This study did not exclude the possibility that participants could suffer from the borderline personality trait that is associated with emotional dysregulation [85] and contributes to work-related stress. Although this study reported the physical and psychological health of participants, we did not explore the specific relationship between common psychiatric conditions such as depression and its relationship with work-related stress. Depression can lead to cognitive impairment and affects work performance [90].

Although this introductory study reveals relatively compact outcomes, and even if it cannot be extended to other situations, it is one of the few studies that discusses personality traits while attempting to integrate aspects of clinical psychology and social psychology. In fact, such studies can be counted on the fingers of one hand and are definitely not part of the mainstream literature.

Furthermore, it may be useful to start research protocols that allow for the application of probabilistic sampling techniques and that use research designs for non-equivalent groups. Future research should move in this direction.

## Figures and Tables

**Table 1:** Correlation between the Organizational and Psychosocial Risk Assessment (OPRA) and the Big Five Questionnaire (BFQ).

ISR (B) and MPH (C)	Extraversion	Agreeableness	Conscientiousness	Neuroticism	Openness
B1 culture and organization	0.231 **	0.204 **	0.233 **	0.253 **	0.102
B2 career development	−0.331 **	−0.276 **	−0.250 **	−0.290 **	−0.213 **
B3 role	−0.274 **	−0.333 **	−0.222 **	−0.376 **	−0.260 **
B4 autonomy	0.218 **	0.194 **	0.251 **	0.207 **	0.186 **
B5 work-family interface	−0.119 *	−0.153 **	−0.048	−0.288 **	−0.185 **
B6 environment and safety	0.180 **	0.130 *	0.196 **	0.236 **	0.099
B7 workload	−0.137 *	−0.184 **	−0.062	−0.242 **	−0.126 *
B8 working time	−0.019	−0.038	0.046	−0.140 *	−0.038
B9 quality of relationships	−0.201 **	−0.339 **	−0.196 **	−0.333 **	−0.251 **
C1 physical health	−0.362 **	−0.304 **	−0.161 **	−0.456 **	−0.276 **
C2 psychological health	−0.227 **	−0.225 **	−0.078	−0.365 **	−0.131 *

** The correlation is significant at the 0.01 level. * The correlation is significant at the 0.05 level.

**Table 2:** ANOVA.

BFQ	Difference	Sum of Squares	Gl	Quadratic Mean	F	Sign
Extraversion	between groups	1660.104	2	830.052	9.629	0.000
	within groups	25,430.312	295	86.204		
	total	27,090.416	297			
Agreeableness	between groups	1319.772	2	659.886	7.35	0.001
	within groups	26,484.003	295	89.776		
	total	27,803.775	297			
Conscientiousness	between groups	297.656	2	148.828	1.553	0.213
	within groups	28,267.408	295	95.822		
	total	28,267.408	297			
Neuroticism	between groups	1343.352	2	671.676	8.692	0.000
	within groups	22,795.672	295	77.273		
	total	24,139.023	297			
Openness	between groups	742.508	2	371.254	4.115	0.017
	within groups	26,613.737	295	90.216		
	total	27,356.245	297			

The ANOVA test is significant at the 0.05 level.

**Table 3:** Post hoc comparison.

Multiple Comparisons - LSD							
Dependent Variable	(I) Risk Category	(J) Risk Category	Mean Difference (I-J)	Std. Error	Sig.	95% Confidence Interval	
						Lower Bound	Upper Bound
Extraversion	medium low	medium high	4.364	1.094	0	2.21	6.52
		high	9.552	4.215	0.024	1.26	17.85
	medium high	medium low	−4.364	1.094	0	−6.52	−2.21
		high	5.188	4.233	0.221	−3.14	13.52
	high	medium low	−9.552	4.215	0.024	−17.85	−1.26
Agreeableness		medium high	−5.188	4.233	0.221	−13.52	3.14
	medium low	medium high	3.867	1.116	0.001	1.67	6.06
		high	8.703	4.301	0.044	0.24	17.17
	medium high	medium low	−3.867	1.116	0.001	−6.06	−1.67
		high	4.836	4.319	0.264	−3.66	13.34
	high	medium low	−8.703	4.301	0.044	−17.17	−0.24
		medium high	−4.836	4.319	0.264	−13.34	3.66
Conscientiousness	medium low	medium high	1.869	1.153	0.106	−0.4	4.14
		high	−2.242	4.444	0.614	−10.99	6.5
	medium high	medium low	−1.869	1.153	0.106	−4.14	0.4
		high	−4.111	4.462	0.358	−12.89	4.67
	high	medium low	2.242	4.444	0.614	−6.5	10.99
		medium high	4.111	4.462	0.358	−4.67	12.89
Neuroticism	medium low	medium high	3.792	1.035	0	1.75	5.83
		high	9.558	3.99	0.017	1.7	17.41
	medium high	medium low	−3.792	1.035	0	−5.83	−1.75
		high	5.766	4.007	0.151	−2.12	13.65
	high	medium low	−9.558	3.99	0.017	−17.41	−1.7
		medium high	−5.766	4.007	0.151	−13.65	2.12
Openness	medium low	medium high	3.2	1.119	0.005	1	5.4
		high	2.364	4.312	0.584	−6.12	10.85
	medium high	medium low	−3.2	1.119	0.005	−5.4	−1
		high	−0.836	4.33	0.847	−9.36	7.69
	high	medium low	−2.364	4.312	0.584	−10.85	6.12
		medium high	0.836	4.33	0.847	−7.69	9.36
